# Five year results of the first ten ACL patients treated with dynamic intraligamentary stabilisation

**DOI:** 10.1186/s12891-016-0961-7

**Published:** 2016-02-27

**Authors:** Stefan Eggli, Christoph Röder, Gosia Perler, Philipp Henle

**Affiliations:** Department of Knee Surgery and Sports Traumatology, Sonnenhof Orthopaedic Center, Berne, Switzerland; Institute for Evaluative Research in Medicine, University of Berne, Berne, Switzerland

**Keywords:** Augmentation, ACL healing, ACL repair, DIS, Ligamys, ACL preservation

## Abstract

**Background:**

In recent years, the scientific discussion has focused on new strategies to enable a torn anterior cruciate ligament (ACL) to heal into mechanically stable scar tissue. Dynamic intraligamentary stabilization (DIS) with LigamysTM was first performed in a pilot study of 10 patients. The purpose of the current study was to evaluate the five year results of this group.

**Methods:**

Inclusion criteria were an ACL rupture not older than 14 days, patient age <45 years, no previous surgery on the injured knee, and regular participation in sports requiring pivoting of the knee joint. Ten consecutive patients (eight males, two females) underwent surgery between August 2009 and February 2010. They were treated by DIS employing an internal stabilizer to keep the unstable knee in a posterior translation, combined with microfracturing and platelet-rich fibrin induction at the rupture site to promote self-healing. Postoperative clinical outcome [Tegner, Lysholm, International Knee Documentation Committee (IKDC), visual analogue scale patient satisfaction score] and assessment of knee laxity was performed at 3, 6, 12, 24 and 60 months.

**Results:**

Median patient age at time of surgery was 23.3 years (range 19–41 years). The median time to surgery was 10 days (range 5–13 days). The rupture was located in the middle third of the ligament in seven patients and in the proximal third in three patients. Eight patients showed additional meniscal lesions, which were surgically treated in six patients. Eight of the ten patients reached the five-years follow-up. Median Lysholm score was 100 (range 90–100); the IKDC score was 98.9 (range 79.3–100); Tegner score was 5.5 (range 5–7); median Lachman difference to the other side was 2 mm (range 0–4 mm). Median patient satisfaction was 10 points (range 8–10 pts.). Four of the ten patients underwent metal removal (tibial implant component) after ACL healing and a consequently stable knee joint. Two patients suffered from a re-rupture at 5 months and 4.2 years after surgery and were treated with a bone-tendon-bone ACL graft.

**Conclusions:**

Dynamic intraligamentary stabilization in ten active patients with a fresh ACL rupture showed a 5-years survival rate of 80 %. At the last follow-up all patients with a functionally healed ACL showed excellent outcomes and satisfaction with regards to the treatment result.

## Background

Rupture of the anterior cruciate ligament (ACL) is the most common knee injury requiring surgical repair [1]. While a conservative treatment approach leads to satisfactory results in a population that places low demand on the knee joint [2, 3], persisting instability prevents patients from participating in activities that require high levels of joint pivoting, such as soccer and skiing. Therefore reconstruction of the the ACL with a tendon autograft has become the gold standard in treating active patients. Despite advances in arthroscopic ACL reconstruction and further developments in surgical techniques results are not in all cases satisfactory and long-term outcome after ACL ruptures - even when treated properly - remains an issue. Biau’s meta-analysis [4] revealed that only about 40 % of patients made a full recovery after ACL reconstruction, with only 33 % having a normal IKDC score after a semitendinosus transplant and 41 % having a normal IKDC after a BTB (ligamentum patellae) transplant. Possible explanations for these outcomes are removal of the native ACL tissue containing sensory nerve fibres resulting in a functional loss of the ligament with regards to its function within the joint’s ‘proprioceptive envelope’, thus impairing muscular stabilization of the knee [5, 6]. Based on this theory, the authors started to investigate a strategy called Dynamic Intraligamentary Stabilization DIS for preserving the torn ACL in 2007.

**Table 1:** Clinical scores, ap-laxity and patient satisfaction at follow-up examinations (median values and range)

Measure	Before injury	3 months	6 months	1 year	2 years	5 years
Lysholm	100 (100–100)	99 (95–100)	100 (95–100)	100 (100–100)	100 (100–100)	100 (90–100)
IKDC	100 (100–100)	94 (89–98)	94 (93–99)	98 (97–100)	100 (97–100)	99 (79–100)
Tegner	6 (4–9)	5 (3–7)	5 (3–8)	6 (4–9)	6 (4–9)	5.5 (5–7)
Δ Lachmann [mm]	5^a^ (3–6)	0 (−4–3)	1 (−3–3)	0 (−2–3)	1 (−1–3)	2 (0–4)
Patient Satisfaction [VAS]	n/a	10 (9–10)	10 (9–10)	10 (10–10)	10 (10–10)	10 (8–10)

^a^After injury, before surgery

**Table 2:** Patient details and course of IKDC scores (median values)

Patient #	Gender	Age at surgery	Occupation	Accident mechanism	pre-op	3 m	6 m	1y	2y	5y
1	m	24	Light physical labour	Soccer	100	90	93	97	97	100
2	m	35	Office	Beachvolleyball	100	94	95	98	97	100
3	m	29	Light physical labour	Judo	100	95	99	100	100	-
4	m	40	Heavy physical labour	Jump off ramp	100	94	94	98	98	98
5	m	23	Heavy physical labour	Bicycle accident	100	95				
6	m	20	Heavy physical labour	Soccer	100	89	94	98	100	100
7	f	19	Heavy physical labour	Alpine skiing	100	98	99	100	100	100
8	m	23	Office	Alpine skiing	1000	95	94	100	100	97
9	f	22	Heavy physical labour	Athletics	100	92	-	97	100	79
10	m	19	Heavy physical labour	Motorcycle accident	100	-	-	-	-	87

The goal of this study was to summarize the data on 10 patients treated with DIS over a period of 5 years follow-up.

## Methods

### Surgical technique

Summarized briefly, the tibial remnants of the torn ACL are guided to the femoral footprint by transosseous resorbable sutures (anatomical reduction). After extensive microfracturing at the femoral footprint and the knee is thans stabilized with a polyethylene cord which is brought under tension by a spring implant (Ligamys™, Mathys Ltd Bettlach, Switzerland) hosted on the anteromedial aspect of the tibia. Thus, the proximal tibia is pulled in a constant posterior drawer position. The spring mechanism allows a dynamic excursion of 8 mm [7], ensuring a continuous tension of the cord over the entire range of motion, as well as to compensate for an anisometric placement of the intraarticular entry points.

### Patients

For inclusion patients had to meet the following criteria: ACL rupture not older than 14 days, patient age <45 years, no previous surgery on the affected knee, and regular participation sports activities. Ten consecutive patients (eight males, two females) underwent the surgical procedure between August 2009 and February 2010. Median age was 23.3 years (range 19–41 years); there were 7 right and 3 left knees. The median time to surgery was 10 days (range 5–13 days). The rupture was located in the middle third of the ligament in seven patients and in the proximal third in three patients. Eight patients showed additional meniscal lesions, of which six were surgically treated.

### Outcome measure

Postoperative clinical outcome [Tegner, Lysholm, International Knee Documentation Committee (IKDC), visual analogue scale for patient satisfaction score] and assessment of knee laxity was performed at 6 weeks, 3, 6, 12, 24 and 60 months. Based on the instruments’ outcome scores a combined success definition was applied. The patients’ preoperative scores were assessed as early as possible, but naturally after the trauma. Knee laxity was assessed by measuring anterior tibial translation at 30 ° flexion with an arthrometer (Rolimeter, Aircast, Neubeuern, Germany) and comparing it with the contralateral knee. All patients were informed that their treatment and follow-up data would be recorded in a scientific database for evidence generation and postmarket surveillance of Ligamys™ and its outcomes, for which they gave their voluntary written informed consent. The Cantonal Ethics Committee of Berne, Switzerland approved on the whole study including patient information and consent, surgical procedure, data collection and analysis (Ref.-Nr. KEK-BE: 048/09).

### Available data and statistical analysis

To express the variability and distribution of the underlying data, the median values of outcome scores and their range were calculated and reported. No inferential statistics were used in this exploratory descriptive study (*n* = 8). There were nine 3-month follow-ups; seven 6-month follow-ups; eight 12-month follow-ups; eight 24-month follow-ups; and eight 5 year follow-ups. The mean interval of the last follow-up of the 8 patients with intact ACL was 60.3 months.

## Results

### Functional results

The median Lysholm score was 100 before injury, 99 (95–100) after 3 months, 100 after 12 months, 100 after 24 months and 100 (90–100) after 5 years. The median IKDC score reached 100 (97–100) after 24 months, and 98.9 (79.3–100) after 5 years. With 5.5 points the group’s median Tegner activity score remained almost the same as before the accident. After 3 months, the median anterior translation difference was 0 mm (−3 to 4) compared with the contralateral side, 1 mm (−1 to 3) after 24 months and 2 mm (0 to 4) after 60 months. Before surgery, it was 5 (range 3–6 mm). Median patient satisfaction was 10 (9–10) after 3 months, 10 (10–10) after 24 months and 10 (8–10) after 5 years (Table 1).

When a combined success definition was applied (AP translation difference ≤3 mm, Lysholm score >84 points [8], IKDC score >84 % [9]), 6 patients fulfilled all 3 criteria at the last follow-up. The distribution of the individual success criteria was as follows: AP translation ≤3 mm, 7/8 patients; Lysholm score >84 points 8/8 patients; and IKDC score >84 %, 7/8 patients (Table 2).

### Complications

At 5 months after surgery, one patient (a 24-year-old male sports student) suffered from a re-rupture after sustaining a direct rotation trauma playing soccer against his surgeon’s recommendation. Until then, the patient had been pain-free at the 3 month follow-up, with a Lysholm score of 97, a Tegner score of 5 and a Lachman difference of 3 mm compared with the opposite side. 6 weeks after the second trauma his completely re-ruptured ACL was replaced by a bone-tendon-bone (BTB) graft. A second re-rupture occurred in a patient 4 years and 2 months postoperative during judo practice with a 110 kg opponent. His last follow-up at two years after surgery had shown no pain and no swelling with Lysholm and Tegner scores of 100 and a Lachman difference of 1 mm compared with the opposite side. He was treated with a BTB graft and a meniscus suture one week after the re-rupture. The two year results of the nine patients with a healed ACL were already published [9].

Four patients had the implant removed between 2 and 11 months after the index surgery (mean 6.5 months) based on a healed ACL and stable knee joint. Reasons were immobility in two cases, pain in one case, and patient desire in one case.

### Patient activity

It becomes visible that most patients were in Tegner classes 5 and 6 before the injury and return to these levels after DIS. Only few patients from higher and lower activity classes need several years to return to their pre-injury levels, or, in case of the most active patient with a pre-injury Tegner class of 9, will eventually end up in lower activity classes.

### Objective stability

The additional anterior translational laxity of the injured knee ranged from 3 to 6 mm before surgery compared to the contralateral knee. After five years, this range was reduced to 0–2 mm. Only one patient had a sudden increase in translation to 4 mm more than the opposite knee, whereby he had previously been at a very good difference of only 1 mm additional laxity. Table 5 shows absolute ap-laxity values (mm) of the injured and the opposite knee before surgery and the preoperative and postoperative differences. Positive differences mean that the affected knee was more lax than the opposite knee.

### Patient satisfaction

The vast majority of patients had satisfaction levels between 9 and 10 very early on and they remain stable until 5 years after surgery in most cases.

## Discussion

The outcomes of the first ten patients ever treated with DIS (LigamysTM implant) are presented in the current case series. Overall function, knee stability and patient satisfaction showed very good and stable results in all cases with a healed ACL. Two patients suffered from a re-rupture, both with an adequate trauma mechanism. In all other cases, excellent function scores were reached within 3–6 months and were maintained until the last follow up 5 years after the index surgery. Four implant removal have been performed and it has to be discussed if this secondary intervention has to be rated as a major downside for the patient. The implant removal is a simple procedure of approximately 5 min that can be done in local anesthesia. Many patients express their explicit will to perform this intervention even when they are not having symptoms linked to the implant. So in our eyes the hardware removal can be seen more as an option for their patient rather than a complication of the index surgery.

The small sample size and the lack of a control group are the major drawbacks of the presented analysis. Therefore a representative failure rate cannot be concluded from this small case series. Analysis of a larger cohort revealed an overall revision rate of 4 % after two years [10]. Results published by other groups outside the author’s institution confirm these findings [11].

Primary repair of the torn ACL has been widely abandoned after arthroscopic ACL reconstruction became popular in the early 1990ies. Important studies by Feagin [12] and Engebretsen [13] showed inferior clinical results after suture repair of the torn ACL. Nevertheless, other authors reported success when limiting the indication to selected cases [14].

Maintaining proprioception of the knee is an essential goal of ACL repair, as compared with reconstruction. Protecting the ACL suture with simultaneously allowing full range of motion and full weight bearing directly after surgery is the major innovation with Dynamic intraligamentary stabilization. The second major advantage of the DIS treatment is the possibility of an early intervention, within days after the trauma. This allows an optimal treatment of concomitant injuries to menisci and/or cartilage which in turn might positively affect long-term outcome after ACL rupturs.

## Conclusions

Dynamic intraligamentary stabilization in the first ten patients with an active lifestyle and a fresh ACL rupture showed a 5-years survival rate of 80 %. At the last follow-up all patients showed excellent function and satisfaction with regards to the treatment result. Given that surgical technique, implant and instrumentation as well as our experiences with healing the ruptured ACL have significantly increased in the last 5 years, this technique might offer an additional option in the treatment of acute ACL injuries.

## Abbreviations

ACL: anterior cruciate ligament
BTB: bone-patellar tendon-bone
DIS: Dynamic Intraligamentary Stabilization
IKDC: International Knee Documentation Committee
